# *DACH1*, a Zona Glomerulosa Selective Gene in the Human Adrenal, Activates Transforming Growth Factor-β Signaling and Suppresses Aldosterone Secretion

**DOI:** 10.1161/HYP.0000000000000025

**Published:** 2015-04-08

**Authors:** Junhua Zhou, Lalarukh Haris Shaikh, Sudeshna G. Neogi, Ian McFarlane, Wanfeng Zhao, Nichola Figg, Cheryl A. Brighton, Carmela Maniero, Ada E.D. Teo, Elena A. B. Azizan, Morris J. Brown

**Affiliations:** From the Clinical Pharmacology Unit, Department of Medicine (J.Z., L.H.S., C.A.B., C.M., A.E.D.T, E.A.B.A., M.J.B.), Cardiovascular Division, Department of Medicine (N.F.), University of Cambridge, Addenbrooke’s Hospital, Cambridge, United Kingdom; Department of Clinical Biochemistry, GenomicsCoreLab, Cambridge NIHR BRC, Department of Clinical Biochemistry (S.G.N., I.M.), and Human Research Tissue Bank, Cambridge University Hospitals NHS Foundation Trust (W.Z.), Addenbrooke’s Hospital, Cambridge, United Kingdom; and Department of Medicine, Faculty of Medicine, The National University of Malaysia (UKM) Medical Centre, Kuala Lumpur, Malaysia (E.A.B.A.).

**Keywords:** aldosterone, DACH1 protein, human, hyperaldosteronism, transforming growth factor beta, zona glomerulosa

## Abstract

Common somatic mutations in *CACNAID* and *ATP1A1* may define a subgroup of smaller, zona glomerulosa (ZG)–like aldosterone-producing adenomas. We have therefore sought signature ZG genes, which may provide insight into the frequency and pathogenesis of ZG-like aldosterone-producing adenomas. Twenty-one pairs of zona fasciculata and ZG and 14 paired aldosterone-producing adenomas from 14 patients with Conn’s syndrome and 7 patients with pheochromocytoma were assayed by the Affymetrix Human Genome U133 Plus 2.0 Array. Validation by quantitative real-time polymerase chain reaction was performed on genes >10-fold upregulated in ZG (compared with zona fasciculata) and >10-fold upregulated in aldosterone-producing adenomas (compared with ZG). *DACH1*, a gene associated with tumor progression, was further analyzed. The role of *DACH1* on steroidogenesis, transforming growth factor-β, and Wnt signaling activity was assessed in the human adrenocortical cell line, H295R. Immunohistochemistry confirmed selective expression of DACH1 in human ZG. Silencing of *DACH1* in H295R cells increased *CYP11B2* mRNA levels and aldosterone production, whereas overexpression of *DACH1* decreased aldosterone production. Overexpression of *DACH1* in H295R cells activated the transforming growth factor-β and canonical Wnt signaling pathways but inhibited the noncanonical Wnt signaling pathway. Stimulation of primary human adrenal cells with angiotensin II decreased *DACH1* mRNA expression. Interestingly, there was little overlap between our top ZG genes and those in rodent ZG. In conclusion, (1) the transcriptome profile of human ZG differs from rodent ZG, (2) *DACH1* inhibits aldosterone secretion in human adrenals, and (3) transforming growth factor-β signaling pathway is activated in *DACH1* overexpressed cells and may mediate inhibition of aldosterone secretion in human adrenals.

## Abstract 2

Supplemental Digital Content is available in the text.

Aldosterone-producing adenomas (APA) were first described 60 years ago by Dr Jerome W. Conn.^1^ Since then improvements in diagnostic techniques have led to some agreement that they may be present in 5% of all hypertensive patients.^2^ The physiological and pathological processes that regulate aldosterone production (and structure of the adrenal gland itself) vary among species. Although APAs themselves might be thought a useful human model, most APA cells morphologically resemble the foamy appearance of the cortisol-producing zona fasciculata (ZF) cells rather than the thin, compact zona glomerulosa (ZG) cells from which aldosterone is physiologically produced.

Recently, however, the existence of heterogeneity among APAs, with ZG-like tumors being commoner than previously thought, has been emphasized by the discovery of somatic mutations, initially in *KCNJ5*, in generally larger APAs with classical ZF-like features^3,4^ and subsequently in other genes (*CACNA1D*, *ATP1A1*, and *CTNNB1*), which we had found in APAs selected for ZG-like features.^5^ Subsequent microarray comparing APAs with the new versus *KCNJ5* mutations revealed significant differences in transcriptome, with expression of some genes varying by >20-fold.

In this study, we sought clues to the origin of APAs through finding previously unsuspected genes that may regulate aldosterone synthesis or other ZG cell functions. We undertook a further microarray comparing APA with ZG and ZF with the objective of finding signature ZG genes. Two thirds of our adrenals came from patients with an APA, of which half contained a *KCNJ5* somatic mutation; the remaining adrenals were from patients with a phaeochromocytoma.

Multiple unsuspected genes were found to be many-fold upregulated in human ZG. We prioritized for further study those whose protein product was shown to be selectively expressed in ZG. Among these, the dachshund homolog 1 gene, *DACH1*, stood out as a candidate regulator of ZG cell function because not only was it >10-fold upregulated in ZG, but its exome was found to have previously undocumented copy number variants (CNVs) in the germline DNA from several patients with APA. Its previously documented roles as a cell determination factor, mediator of steroid responses in hormone-sensitive tumors,^6–8^ and possible links with the transforming growth factor-β (TGF-β) and Wnt signaling pathways all provided further biological reason to investigate the physiological and pathological role of *DACH1* in the adrenal. We report that *DACH1* increases TGF-β signaling and decreases aldosterone production.

## Methods

### Microarray Assay

Microarray assay was performed using the Affymetrix Human Genome U133 Plus 2.0 Array by GenomicsCorelab, Cambridge. Fifty-six RNA samples acquired through laser capture microdissection were assayed—14 trios of ZF, ZG, and APA from patients with Conn’s syndrome and a further 7 pairs of ZF and ZG adjacent to a pheochromocytoma. Seven of the APAs contained a somatic mutation in *KCNJ5*, whereas the remaining 7 were wild-type. Microarray results were validated by quantitative real-time polymerase chain reaction. Further details are provided in the online-only Data Supplement.

### Immunohistochemistry

Commercial DACH1 antibody (catalog, HPA012672; Sigma, United Kingdom; 1:200 dilution), KCNJ5 antibody (catalog, HPA017353; 1:100 dilution), PCP4 antibody (catalog, HPA005792; 1:200 dilution), custom made CYP11B1 (Severn Biotech Ltd, United Kingdom), and CYP11B2 antibody (a kind gift from Dr Celso E. Gomez-Sanchez) were used as the primary antibodies. Negative controls, whereby primary antibodies were omitted, resulted in a complete absence of staining. Images were captured using a standard bright field microscope, U-TV1-X digital camera, and the CellD software (Olympus Ltd, United Kingdom).

### Cell Culture Experimentation

The human adrenocortical carcinoma cell line, H295R, was cultured in growth medium consisting of supplemented Dulbecco Modified Eagle Medium/Nutrient F-12 Ham as previously described.^5^ For short interfering RNA experiments, on-target plus human *DACH1* short interfering RNA smart pool (Thermo Scientific, Waltham) was used. For overexpression experiments, cells were transfected with *DACH1* 706aa, *DACH1* 709aa, or vector control plasmids using Amaxa® Cell Line Nucleofector® Kit R (Lonza, Switzerland). Further details are provided in the online-only Data Supplement.

### Signaling Pathway Activity Assay

TGF-β signaling and canonical Wnt signaling were quantified using the Cignal SMAD Reporter (luc) Kit (SABiosciences, Madison) and Cignal TCF/LEF Reporter (luc) Kit (SABiosciences) respectively. Noncanonical Wnt signaling was measured using an AP-1 reporter construct that was created by cloning at the KpnI and XhoI sites an oligo containing 7 copies of the AP-1 binding element (TGACTAA) into the luciferase construct pGL4.10(luc2) (Promega, Madison). Further details are provided in the online-only Data Supplement.

### Data Analysis

Results are expressed as mean values with SEM and compared using the 2-sided Student *t* test. The significance level of *P*<0.05 was considered to indicate statistical significance. Statistical analysis was performed using standard statistical software.

## Results

### Clinical Features of Patients with Conn’s Syndrome Involved in the Microarray Assay

The average age of the patients was 45.6 years, with a 1:1 ratio of women to men. Postadrenalectomy, systolic blood pressure, diastolic blood pressure, and plasma aldosterone decreased, whereas serum potassium and plasma renin increased confirming that the patients had primary aldosteronism (Table S1 in the online-only Data Supplement). Seven APAs contained a somatic *KCNJ5* mutation, whereas the remaining 7 were wild-type with some ZG-like features (high percentage of compact cells; low *CYP17A1* expression; Table S2).

### Microarray Result

**Figure 1.**
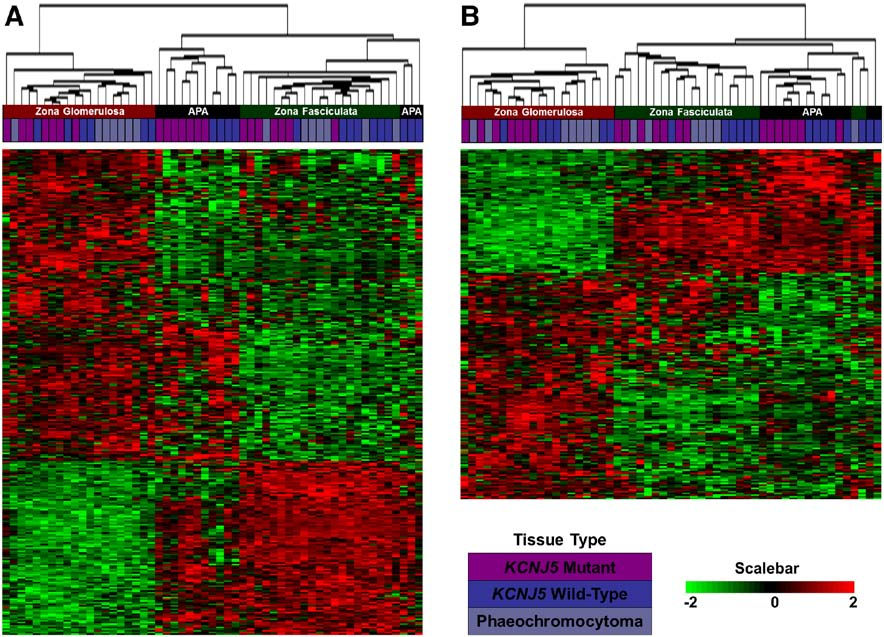
The heat map and dendrogram of unsupervised clustering of (**A**) 293 genes differentially expressed in zona glomerulosa (ZG) compared with zona fasciculata (ZF) and (**B**) 210 genes differentially expressed in aldosterone-producing adenoma (APA) compared with ZG. Unsupervised cluster analysis separated ZG, ZF, and APA from patients with a *KCNJ5* somatic mutation from those without. Red and green indicate high and low expression, respectively. The purple, blue, and grey boxes in the dendogram indicate the tissue came from a patient with Conn’s syndrome with a somatic *KCNJ5* mutation in their APA, without a *KCNJ5* mutation, or from a patient with pheochromocytoma, respectively.

Among the 22 148 genes, 293 genes were differentially expressed between ZG and ZF (fold change>2; *P*<1.0×10^–4^; Figure 1A). The top 50 upregulated genes are listed in Table S3. Confirming the correct selection of ZG tissue was the expected high ZG:ZF ratio for the transcription factor gene *NR4A2*, a known selective ZG gene. Quantitative real-time polymerase chain reaction for *CYP11B1*, *CYP11B2*, and *CYP17A1* expression levels in ZF and ZG also confirmed sample selection of each zones. However, none of the top 6 genes, which showed >10-fold higher expression in ZG than ZF, was expected (*LGR5*, *VSNL1*, *ANO4*, *NEFM*, *VCAN*, and *DACH1*; Table 1). Quantitative real-time polymerase chain reaction validated the microarray results for all of these, with generally higher selectivity for ZG than on the microarray (Table 1). Unsupervised cluster analysis of the 293 genes largely separated ZG, ZF, and APA of patients with a *KCNJ5* somatic mutation from those without (Figure 1A).

**Table 1.**
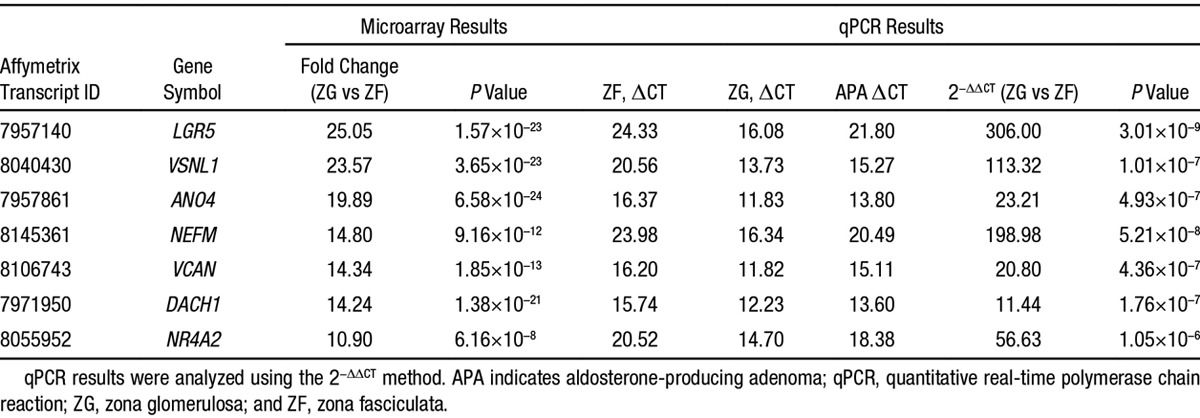
Upregulated Genes in ZG, Compared With ZF (Fold Change, >10)

In the comparison of APA with ZG, 210 genes were differentially expressed (fold change>2; *P*<1.0×10^–4^; Figure 1B). Again, unsupervised cluster analysis of these 210 genes mostly separated ZG, ZF, and APA from patients with a *KCNJ5* somatic mutation from those without (Figure 1B). The top 50 of those upregulated genes are listed in Table S4. Interestingly the 2 genes with fold change>10 are *SULT2A1*, the sulfotransferase enzyme that sulfates adrenal steroid, and *MC2R*, the ACTH receptor (Table 2). This finding is in agreement with previous microarray reports documenting high expression of these genes in APAs.^9^

**Table 2.**
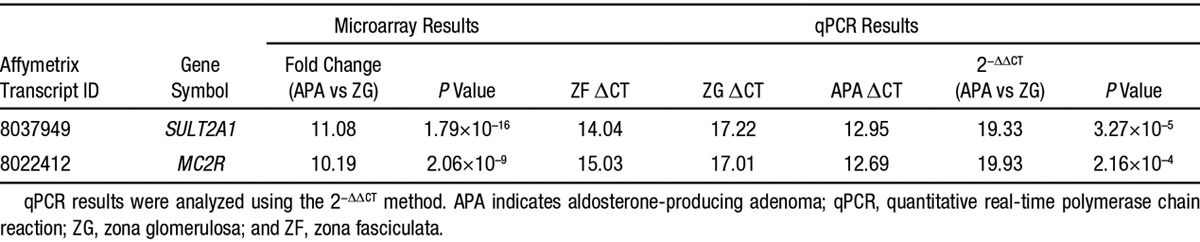
Upregulated Genes in APA, Compared With ZG (Fold Change, >10)

### DACH1 Variant Sequences

CNVs of glycine-repeat deletions in *DACH1* discovered by our previous exome sequencing were validated and confirmed by Sanger Sequencing of the DNA from APAs and the adjacent adrenal glands (AAG) and from the blood of both patients with Conn’s syndrome and healthy controls (Table S5). These CNVs have not been reported on the National Institutes of Health server of 8000 exomes. The different number of glycine deletions, in the region from chromosome 13: 72 440 660 to 72 440 689, translates into DACH1 proteins between 703aa and 708aa in length. The heterozygote of 705aa and 706aa was commoner than the homozygote wild-type 706aa (Figure S1). We did not detect any significant difference in frequency of CNVs between patients with Conn’s syndrome and controls.

### DACH1 Protein Expression and Colocalization With Other ZG/ZF Proteins

**Figure 2.**
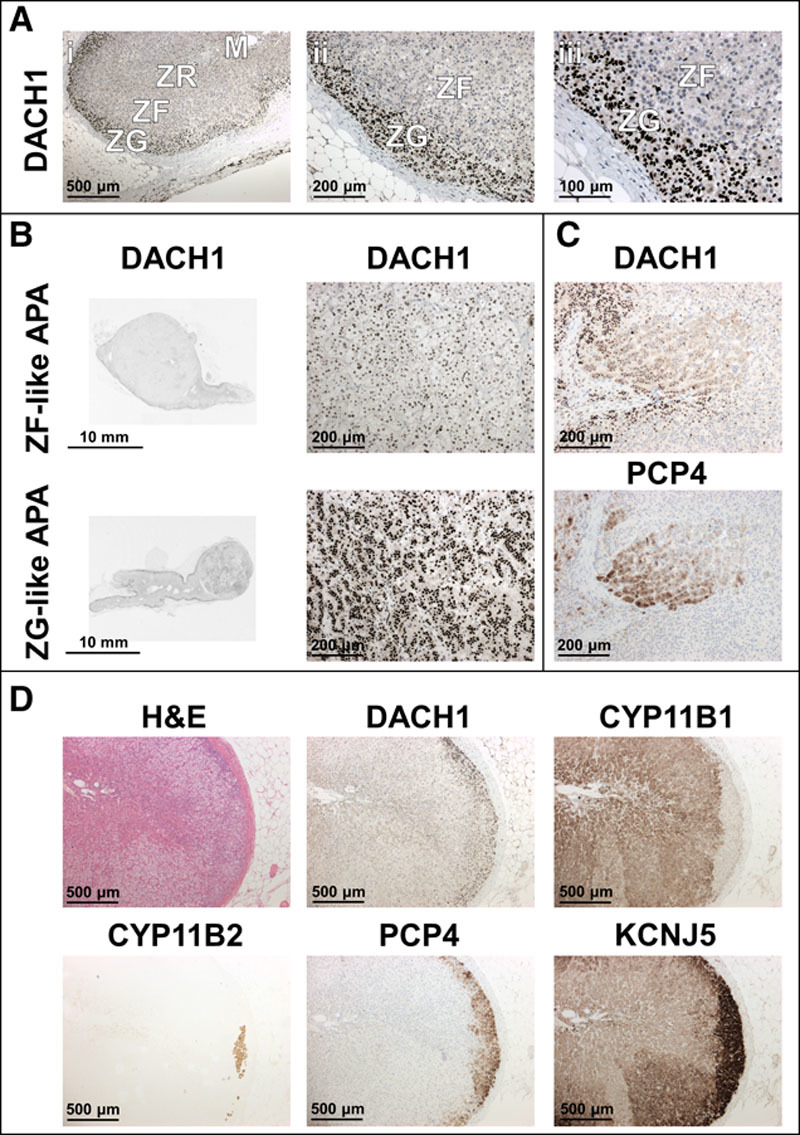
Immunohistochemistry (IHC) of DACH1 in formalin-fixed paraffin-embedded human adrenal sections. **A**, IHC of DACH1 in adjacent adrenal glands. DACH1 is highly expressed in zona glomerulosa (ZG) cell’s nuclei. Pictures are representative of 13 adrenals from 10 patients with aldosterone-producing adenomas (APA) and 3 with pheochromocytomas. **B**, IHC of DACH1 in APAs. ZG-like APAs appeared to have similar staining of DACH1 to their adjacent zona glomerulosa. **Left**, Scanned images. **Right**, Zoomed insets of left panel. **C**, Colocalization of DACH1 cytoplasmic staining with PCP4 in cell clusters. **D**, Hematoxylin and eosin (H&E) stain and IHC of DACH1, CYP11B1 (as a zona fasciculata [ZF] marker), and ZG markers, CYP11B2, PCP4, and KCNJ5, in serial adrenal sections from a patient with Conn’s syndrome.

DACH1 protein expression in the normal adrenal glands adjacent to APA or phaeochromocytoma was highly selective for ZG compared with other adrenal zones, with the staining being confined to nuclei (Figure 2A; Figure S2A). There was similar nuclear staining of APAs (Figure 2B). There were also clusters of cells with mixed DACH1 nuclei and cytoplasmic staining deep within the adrenal cortex away from the capsule, which also stained for a previously documented ZG marker, PCP4^10^ (Figure 2C; Figure S2B and S2C). The distribution of DACH1 appeared the most diffuse but ZG-selective of all the ZG markers, CYP11B2, KCNJ5, and PCP4 (Figure 2D; Figure S3). As previously noted,^11^ CYP11B2 itself was patchy in the ZG.

### DACH1 Inhibits Aldosterone Production

**Figure 3.**
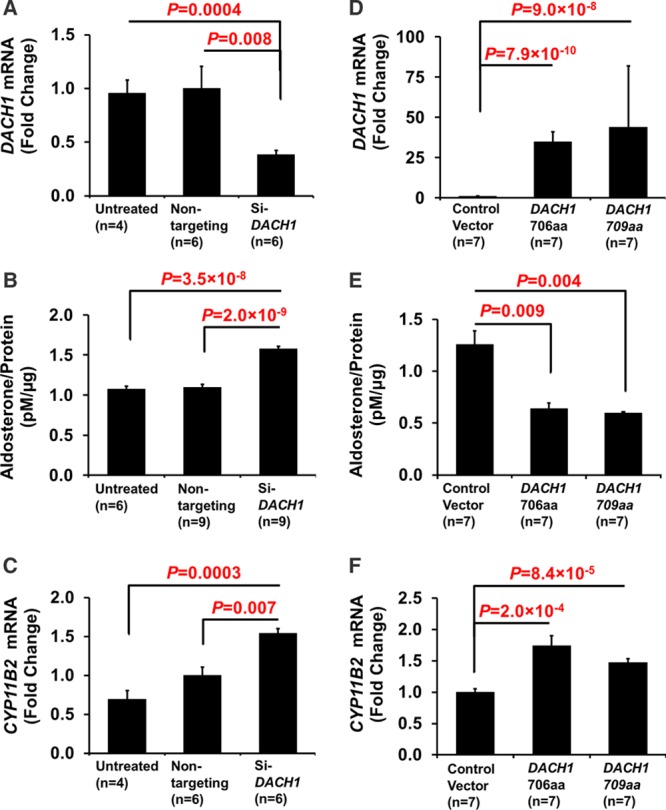
Effect of *DACH1* on aldosterone production. Silencing of *DACH1* in H295R cells increased aldosterone production compared with control (nontargeting), whereas overexpression of *DACH1* decreased aldosterone production compared with control vector. **A**–**C**, H295R cells were treated with vehicle control (untreated), nontargeting siRNA or Si-*DACH1* for 48 h after which 24-h supernatant was collected for aldosterone measurement. **D**–**F**, H295R cells were transfected with either control vector, *DACH1* 706aa or *DACH1* 709aa—24-h supernatant was collected for aldosterone measurement after 48 h. mRNA expression of *DACH1* (**A** and **D**) and (**C** and **F**) mRNA expression of *CYP11B2* after transfection of either siRNAs (**A** and **C**) or overexpression vectors (**D** and **F**).

Silencing of *DACH1* in H295R cells reduced *DACH1* mRNA by 62% compared with their nontargeting short interfering RNA control (*P*=0.008; Figure 3A). This resulted in a 75% reduction of protein levels (*P*=0.009; Figure S4A). Aldosterone secretion from Si-*DACH1* H295R cells was increased (143%; *P*=2.0×10^–9^; Figure 3B), in association with a similar increase in *CYP11B2* mRNA levels (154%; *P*=0.007; Figure 3C). Overexpression of *DACH1* (Figure 3D; Figure S4B) decreased aldosterone production to 51% of vector control (*P*=0.009; Figure 3E). Surprisingly, this was accompanied by a 174% increase in *CYP11B2* mRNA levels (*P*=2.0×10^–4^; Figure 3F).

### Stimulation of Primary Human Adrenal Cells With Angiotensin II Downregulates DACH1

Angiotensin II stimulation downregulated *DACH1* mRNA expression to 54% in primary human adrenal cells (*P*=0.002; Figure S5A). This mRNA change was accompanied with the expected upregulation of *CYP11B2* to 1891-fold (*P*=0.002; Figure S5B) and elevation of aldosterone to 2.4-fold (*P*=0.0002; Figure S5C).

### DACH1 Regulates the Activity of the TGF-β and Wnt Signaling Pathways

**Figure 4.**
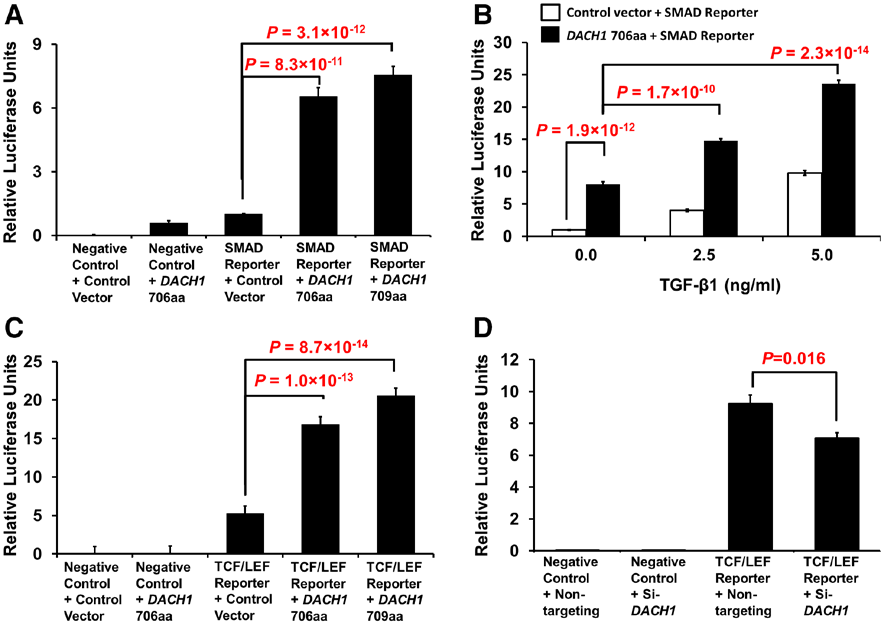
*DACH1* regulates transforming growth factor-β (TGF-β) and Wnt signaling activity. **A**, Overexpression of *DACH1* in H295R cells increased TGF-β signaling activity. H295R cells were cotransfected with either a negative control or a SMAD reporter and with control vector, *DACH1* 706aa or 709aa, to measure *DACH1*’s effect on TGF-β signaling activity; n=10. **B**, The effect of *DACH1* on increasing TGF-β signaling activity in H295R cells was additive to the effect of TGF-β1 exposure. H295R cells were cotransfected with a SMAD reporter and control vector, *DACH1* 706aa or 709aa, and treated with varying TGF-β1 concentration; n=9. **C**, TCF/LEF reporter assay showed upregulation of Wnt signaling activity in H295R cells overexpressing *DACH1*. H295R cells were cotransfected with either a negative control or a TCF/LEF reporter, and with control vector, *DACH1* 706aa or 709aa, to measure *DACH1*’s effect on Wnt signaling activity; n=13. **D**, TCF/LEF reporter assay showed downregulation of Wnt signaling activity in *DACH1*-silenced H295R cells. H295R cells were cotransfected with either a negative control or a TCF/LEF reporter, and with either a nontargeting siRNA or a siRNA specific for *DACH1* (Si-*DACH1*), to measure endogenous *DACH1*’s effect on Wnt signaling activity; n=4.

Overexpression of *DACH1* 706aa and 709aa in H295R cells dramatically upregulated TGF-β signaling activity to 6.5- and 7.6-fold, respectively, of control vector (*P*=8.3×10^–11^ and 3.1×10^–12^, respectively; Figure 4A). The effect of *DACH1* on increasing TGF-β signaling activity was additive to the effect of 2.5 and 5 ng/mL TGF-β1 exposures (*P*=1.7×10^−10^ and 2.3×10^−14^, respectively; Figure 4B). *DACH1* 706aa- and 709aa-overexpressed H295R cells also increased the canonical Wnt signaling activity to 3.2 and 3.9-fold control vector, respectively (*P*=1.0×10^−13^ and 8.7×10^−14^, respectively; Figure 4C). Reversely, silencing of endogenous *DACH1* led to a 22% reduction in activity (*P*=0.016; Figure 4D). The same was not seen with the noncanonical Wnt signaling pathway. Overexpression of *DACH1* 706aa plasmids decreased noncanonical Wnt signaling activity by 61% (*P*=5.2×10^–9^; Figure S6A).

To test whether the results may have been skewed by the presence of the activating S45P β-catenin mutation in the H295R cell line, transient overexpression of *DACH1* was performed in HEK293 cells. Yet again overexpression of both *DACH1* 706aa and 709aa plasmids caused significant increase in canonical Wnt signaling activity (*P*=0.009; Figure S6B).

## Discussion

The human adrenal cortex, like many mammalian adrenals, consists of 3 histologically and functionally different zones: the ZG, ZF, and zona reticularis.^12^ These zones have distinct roles in steroid hormone production, with the ZG-synthesizing mineralocorticoids (aldosterone), the ZF-producing glucocorticoids (cortisol), and the zona reticularis–secreting adrenal androgens (sex steroids).^13–15^ Although differential expression of the key steroidogenic enzymes among the zones is well established and a few other differences have been apparent on in situ hybridization or immunohistochemistry for candidate molecules,^16,17^ no systematic comparison of the transcriptomes in human adrenal has previously been performed. Such a comparison might elucidate how the functional distinctions in physiology arise and are regulated. In addition, the comparison of ZG and ZF transcriptomes might point to the different origin within the adrenal for APA bearing different somatic mutations.

The selective acquisition of ZG and ZF, using cresyl violet staining followed by laser capture microdissection, enabled a comparison of paired transcriptomes, with each other and with adjacent APAs. The results were a surprise (Tables 1 and 2). We identified 293 genes >2-fold differentially expressed in ZG compared with ZF and 210 genes >2-fold differentially expressed in APA compared with ZG, of which most were unsuspected. We had expected to find higher expression in ZG of genes previously associated with aldosterone production. Because we had previously found that APAs lacking *KCNJ5* mutations, with histological features resembling ZG, had a different gene expression profile from *KCNJ5*-mutant ZF-like APAs, we expected to find an overlap with genes upregulated in the ZG when compared with ZF. Although unsupervised cluster analysis did separate ZG, ZF, and APA with a *KCNJ5* mutation from those without (Figure 1), only 9 upregulated ZG genes identified had previously been documented involvement in aldosterone synthesis pathway.^18^ Only 18 of the 50 most upregulated genes in normal ZG were also upregulated (fold change>2) in APAs when using ZF as the comparator tissue.

Of the genes upregulated by >10-fold in ZG when compared with ZF (*LGR5*, *VSNL1*, *ANO4*, *NEFM*, *VCAN*, *DACH1*, and *NR4A2*), only *VSNL1* and *NR4A2* have previously been linked to regulation of aldosterone production.^19,20^ This article reports the studies on *DACH1*. Both microarray and quantitative real-time polymerase chain reaction results showed that the *DACH1* gene expression level was the highest in ZG compared with APAs and ZF (Table 1). This gene encodes for the dachshund family transcription factor 1, a nuclear protein of the cell fate determination pathway involved in Drosophila eye development. *DACH1* is considered a tumor suppressor as loss of *DACH1* expression (which is present in many tissues) has been associated with prostate, endometrial, hepatocellular, breast, lung, colorectal, and ovarian carcinoma.^7,8,21–25^ A study on breast cancer suggested that *DACH1*’s mode of action was negative regulation of TGF-β signaling through binding with Smad4.^26^ Alternatively, a study on colorectal cancer showed that *DACH1* regulated Wnt signaling pathway by phosphorylating β-catenin.^24^ Both of these signaling pathways have previously been associated with aldosterone production.^27,28^ Thus, we focused on the role of *DACH1* in steroidogenesis and its effect on TGF-β and Wnt signaling pathways in human adrenals.

A striking observation of DACH1 in human adrenals was the specific nuclear localization except in a few APAs and PCP4-stained cell clusters where distribution was additionally or solely cytoplasmic. Nuclear distribution of DACH1 has been observed in other tissues with loss associated with apparent decline in tumor suppression. Because of the possible roles of DACH1 in regulation of transcription and tumor suppression, we were intrigued to discover a common 5′ CNV, which proved to be a trinucleotide GGC repeat encoding glycine (Figure S1). Trinucleotide repeat disorders are the most abundant forms of repeat expansion diseases associated with oncological pathologies.^29^ The classic example is the androgen receptor gene polymorphisms, a CAG and a GGC trinucleotide repeats in exon 1, the length of which have been linked to prostate cancer survival and breast cancer risk.^30^ Sequencing across GGC repeats is problematic, and as such, we have yet to compare CNV frequency between primary aldosteronism patients and healthy controls in sufficient numbers. Similarly, generation of constructs of varying GGC repeats proved challenging; in this study, we managed to characterize the activity of 2 variants: the wild-type (*DACH1706aa*), with 8 repeats, and *DACH1 709aa*, with 11 repeats.

Our key finding during functional analyses was that *DACH1* suppresses aldosterone production. This was manifested both as a reduction in aldosterone secretion after overtransfection of H295R cells and an increase in aldosterone secretion after silencing of endogenous *DACH1*. The increase was most probably because of the parallel increase in *CYP11B2* transcription (Figure 3). Complementing the results seen in *DACH1*-silenced H295R cells is the decrease of *DACH1* mRNA expression level in stimulated primary human adrenal cells (Figure S5). The signaling pathway for *DACH1*’s suppression of aldosterone secretion seems most likely to be TGF-β. In the relatively sparse literature currently available, this is the pathway most often associated with *DACH1*, although contradictory conclusions have been obtained with different tissues and cell lines. For instance, restoration of *DACH1* in the liver cell line, SK-Hep1, elevated TGF-β signaling activity,^21^ whereas *DACH1* expressed in breast cancer cell lines inhibited TGF-β–induced apoptosis.^26^ In our human adrenal cell line, H295R, overexpression of *DACH1* increased TGF-β signaling pathway activity as measured by the SMAD reporter (Figure 4A). This is in remarkable agreement with the diffuse, selective expression of TGF-β1 in ZG (and zona reticularis but not ZF) of human adrenal and its potent inhibition of basal, ACTH-, cAMP-, potassium-, and angiotensin II–stimulated aldosterone secretion.^27,31,32^ Although our study is in apparent disagreement with that seen in breast cancer, both studies found *DACH1* to repress AP-1 activity (Figure S6A).

A better recognized signaling pathway in H295R cells and the adrenal in general is the canonical Wnt pathway.^28,33,34^ There is probably crosstalk between TGF-β and Wnt signaling. β-catenin and LEF/TCF, downstream components of the Wnt signaling cascade, have been reported to form a complex with Smad4, an essential mediator of TGF-β signaling pathway.^35^ A recent study reported that silencing of *DACH1* in the human colon cancer cell line, HCT116, leads to an increase in LEF/TCF reporter activity, after addition of TGF-β1, which was reduced after restoration of *DACH1* expression.^27^ We did indeed find a marked, ≈3-fold change in LEF/TCF after *DACH1* transfection of H295R cells, but in the opposite direction to that expected—an increase in transfection and reduction on silencing. As with TGF-β, the effect of *DACH1* on Wnt probably varies between tissues. It must be noted though that both H295R cells and HCT116 cells have a constitutively activating β-catenin mutation, S45P and S45Del respectively. Because canonical Wnt activation is associated with increased aldosterone production,^28^ whereas *DACH1* and TGF-β do the reverse, we reason that TGF-β is likely to be the primary signaling pathway for *DACH1*, with Wnt activation being a secondary or compensatory response. Wnt-driven *CYP11B2* transcription could indeed be the explanation of why, on transfection of *DACH1*, we found an increase in *CYP11B2* mRNA despite reduction in aldosterone secretion. However, Wnt activation and aldosterone synthesis in itself may not be a simple relationship; other adrenocortical adenomas (eg, cortisol secreting adenomas and nonfunctional adenomas) which do not produce aldosterone can also contain constitutively active mutant β-catenin.^36^

In the past decade, many studies have investigated the differential transcriptome profiles between normal adrenals and APAs.^37,38^ However, the common reference samples used were the entire adrenal gland or adrenal cortex.^37,38^ One study in rodents compared the expression profile of ZF and ZG within individual adrenals, using laser capture microdissection similar to ours for sample acquisition.^39^ Their microarray analysis presented a clear transcriptome differences between the 2 cortical zones, ZF and ZG, yet interestingly little overlap between the most upregulated genes in ZG between their study and ours (only 3 of the top 50).^39^ The gross difference in *CYP11B2* expression between the ZG of the 2 species, and our finding that at least 1 of our top upregulated ZG genes inhibits aldosterone secretion, suggests that–unusually for an endocrine gland–upregulated genes may be more concerned with suppressing hormone secretion rather than stimulation. This is corroborated by the paucity of ZG genes in ZG-like APAs, where, by definition, the signaling process is concerned with activation of aldosterone production. A probable unique feature of humans is our chronic high salt intake, resulting effectively in a chronic salt-induced suppression of aldosterone production.

A final pointer to the inhibitor role of *DACH1* comes from our finding of clusters of cells with *DACH1* cytoplasmic staining (not nuclei staining as seen in ZG) that colocalized with *PCP4*, a previously documented ZG marker that is also upregulated in APAs.^10^ *PCP4* is a calmodulin-binding protein that accelerates calcium association and dissociation with calmodulin. In contrast to *DACH1*, silencing of *PCP4* in H295R cells decreased *CYP11B2* and aldosterone production, whereas in APAs, the mRNA levels of *PCP4* positively correlated with those of *CYP11B2*.^10^ Furthermore, the change from purely nuclear to cytoplasmic staining for *DACH1* in some of the clusters resembles that reported in the ovary, at the onset of cancer.^25^

Although this study presents a within-patient comparison of ZG, ZF, and APA transcriptomes, its design leaves some limitations. One is that the spectrum of diagnoses reflects current indications for adrenalectomy in our hospital, and we did not include completely normal adrenals. Secondly, the immortalized adrenal cell line, H295R, is not a perfect model for native ZG cells.^40^ Although our data in primary adrenal cells support *DACH1*’s role in aldosterone regulation, they too are imperfect, being a mixture of ZG, ZF, and zona reticularis cells.

In conclusion, we have defined a series of novel transcript differences between human ZG and ZF and drawn attention to a cell machinery that may be more concerned with inhibition than stimulation of hormone secretion. We propose that the *DACH1*/TGF-β pathway enables ZG to switch off aldosterone production.

### Perspectives

Primary aldosteronism is the most common cause of secondary hypertension of which APAs are responsible for 30% to 50% of cases. Recently, somatic mutations in ion channels (*KCNJ5*, *ATP2B3*, *ATP1A1*, and *CACNA1D*) were found in APAs, with the latter 2 delineating a common ZG subtype of APAs. The discovery of APAs that seem to originate from the ZG of normal adrenal predicted similarity of transcriptome between ZG and such APAs. Instead, we find genes upregulated in ZG that are less abundant or absent in APAs and seem to inhibit hormone production. We consider this an unusual finding for an endocrine gland. We speculate that the inhibition contributes to the well-documented patchiness of aldosterone production in human ZG, which is attributed to salt excess.

## Acknowledgments

We thank Dr Paul D. Upton, Dr Murray Clarke, Prof. Richard G. Pestell, Prof. M. Guo, Prof. C. Wang, and Prof. K. Wu for their assistance on *DACH1* experiments. We thank Prof. Ales Cvekl for providing the *DACH1* 706aa plasmids. We thank R. Kuc and the Human Research Tissue Bank of Addenbrooke’s Hospital, which is supported by the National Institute for Health Research Cambridge Biomedical Research Centre, for help with storage of adrenal tissue.

## Sources of Funding

The work was funded by a National Institute for Health Research (NIHR) Senior Investigator Award (NF-SI-0512-10052) to M.J. Brown, the Wellcome Trust (085687/Z/08/A), and the NIHR Cambridge Biomedical Research Centre (Cardiovascular). J. Zhou and E.A.B. Azizan were supported by The Cambridge Commonwealth, European & International Trust. J. Zhou was also supported by the Sun Hung Kai Properties–Kwoks’ Foundation.

## Disclosures

None.

## Novelty and Significance

### What Is New?

- We report, for the first time, the complete transcriptome of human zona glomerulosa (ZG), zona fasciculata, and adjacent aldosterone-producing adenomas. The key observation is that few of the genes upregulated in ZG were also upregulated in aldosterone-producing adenomas or had previous documented involvement with aldosterone production.

### What Is Relevant?

- Contrary to usual expectation from a gene highly expressed in an endocrine gland, *DACH1* suppresses secretion of aldosterone. We elucidated that the probable signaling pathway for *DACH1*’s inhibition of aldosterone secretion was through the transforming growth factor-β signaling pathway. We showed that *DACH1* overexpression has the same (and additive) effective on transforming growth factor-β signaling in adrenocortical cells as does transforming growth factor-β1 itself.

### Summary

The overall relevance of our findings is that upregulated genes, such as *DACH1*, may be more concerned with suppressing hormone secretion rather than stimulation. This is corroborated by the paucity of ZG genes in aldosterone-producing adenomas, where, by definition, the signaling process is concerned with activation of aldosterone production. In summary, we have defined a series of novel gene expression profile of human ZG and drawn attention to the *DACH1*/transforming growth factor-β pathway that enables ZG to switch off aldosterone production.
